# Influence of Magnetic Field on Sound Transmission Loss of the Unit Filled with Magnetorheological Fluid

**DOI:** 10.3390/ma15176032

**Published:** 2022-09-01

**Authors:** Xiaomei Xu, Yaqin Wang, Yiwei Wang

**Affiliations:** College of Automobile and Traffic Engineering, Nanjing Forestry University, Nanjing 210037, China

**Keywords:** magnetorheological fluid, sound transmission loss, impedance tube, magnetic field

## Abstract

To explore the feasibility of applying magnetorheological fluids (MRFs) in the field of noise control, the influence of the magnetic field intensity and direction on the sound transmission loss (STL) of a unit filled with MRF (MRF unit) were investigated in this study. First, two types of test sample containing the MRF unit were designed and fabricated. The magnetic field applied to the MRF was provided by the permanent magnets used in pairs. The direction of the magnetic field was perpendicular or parallel to the direction of the sound wave propagation. The distribution of the magnetic field intensity of the two types of test samples was simulated using magnetostatic finite element analysis and validated with the magnetic field intensity measured using a Teslameter. For comparison, test samples containing air and water units were also prepared. Then, the STL of the two types of test samples were measured under different magnetic field intensities using the impedance tube method. Finally, the STL curves of the two types of test samples were presented, and the influence of magnetic field intensity and direction on the STL were discussed. The results demonstrate that the magnetic field direction has a significant influence on the STL of the MRF unit. In addition, when the magnetic field direction is parallel to the sound propagation direction, the STL of the test sample containing MRF unit significantly increases with the increase of the magnetic field intensity at low and middle frequencies.

## 1. Introduction

Noise at low and middle frequencies often needs to be suppressed using active noise control (ANC) technology. ANC can effectively reduce noise over a wide frequency range, which usually requires a large power source and complex control system. The continuous emergence of modern smart materials provides an effective approach for reducing noise at low and middle frequencies [1,2].

Magnetorheological fluids (MRFs) are typical smart materials that are considered as ideal controllable damping materials. They exhibit a low viscosity of Newtonian fluids in the absence of magnetic fields and a high viscosity and low fluidity of Bingham fluids under magnetic fields. Moreover, the transition response between their fluid- and solid-like states is quick, and this transition process is reversible [3,4]. Given these advantages, MRFs have been applied in many engineering fields, such as polishing [5], sealing [6], braking [7], vibration control [8,9], etc. In addition to MRF dampers, the vibration-suppressing performance of MRF-based structures under an external magnetic field has been extensively investigated. Liu et al. [10] proposed an impedance modulating structure based on the MRF and studied its vibration transmissibility. The results showed that the modulation of MRF impedance validly showed excellent attenuation for wave propagation. Irazu et al. [11] analyzed the vibrational response of a smart sandwich structure with an MRF layer under an external magnetic field. Wei et al. [12] and Soroor et al. [13] studied the dynamic properties and vibration-suppressing performance of sandwich beams with MRF cores. Eshaghi et al. [14] and Arani et al. [15] investigated the vibration characteristics of a sandwich plate partially treated with MRFs or an MRF core.

Although MRFs have been widely studied for vibration control applications, limited studies have been conducted to investigate their acoustical properties and explore their potential for noise control applications. Polunin et al. [16,17] performed the experimental studies of the acousto-magnetic effect (AME) in a magnetic fluid and investigated the dispersed composition and magnetic and geometrical parameters of magnetic fluid nanoparticles based on the AME. Rodríguez-López et al. [18,19] analyzed the effects of uniformity, direction, and intensity of the magnetic field on the attenuation of ultrasonic elastic waves in MRF, and the results showed that the sound attenuation increased with an increase in the magnetic intensity until saturation was reached. Shen et al. [20] and Baev et al. [21] investigated the acoustic properties of MRFs in an applied magnetic field. Zielinski et al. [22] and Muhazeli et al. [23] investigated the acoustic absorption properties of foams coped with MRFs under magnetic fields, and found that the sound absorption frequency of the foams could be manipulated by varying the magnetic field. Yan et al. [24] fabricated a phononic crystal (PnC) structure comprising periodically arranged hollow pillars filled with MRF, and the results showed that MRF filling could control the frequency range at the bandgap and passband of the PnC plate.

In recent years, the STL of MRF-based sandwich panels has been studied, and their external magnetic fields have mainly been provided by electromagnets. Hemmatian et al. [25,26] fabricated a 0.1 m diameter clamped circular MRF-based sandwich panel to investigate its STL with an anechoic box including two anechoic spaces and the fabricated electromagnet. The results showed that amplifying the magnetic field intensity could cause the natural frequency of the panel to increase linearly, and the STL of the panel at the resonance frequency increased with increasing magnetic field intensity. Mahjoob et al. [27] studied the STL of MRF-based multilayered panels using an impedance tube, and they found that the STL values increased considerably (up to 30 dB) as the field strength increased when the frequency was less than 3 kHz.

Although the acoustic properties of MRFs and the effect of the magnetic field intensity on their STL have been studied in the existing literature, the effect of the magnetic field direction on the STL of MRFs has not been fundamentally investigated. Moreover, the existing related literature mostly adopts the magnetic field provided by electromagnets to study the effects of the magnetic field on the STL. Compared with permanents, although electromagnets can easily adjust the magnetic field and provide more uniform magnetic fields, electromagnets that can obtain high magnetic field intensities are often large and heavy, and they can easily introduce heat to the magnets. However, small electromagnets can only provide a very weak electromagnetic field, which leads to not obvious effect of the magnetic field intensity on the STL. Therefore, the motivations and contributions of this study are to investigate the influence of the magnetic field intensity and direction on the STL of an MRF unit based on the magnetic field provided by permanent magnets, and to explore the feasibility of applying MRFs in the field of noise control at low and middle frequencies.

The remainder of this paper is organized as follows. The two types of test samples are described in the following section. Section 3 presents an experimental study. In Section 4, experimental results are presented and discussed. Finally, concluding remarks and future work are provided.

## 2. Preparation of the Test Samples

**Figure 1:**
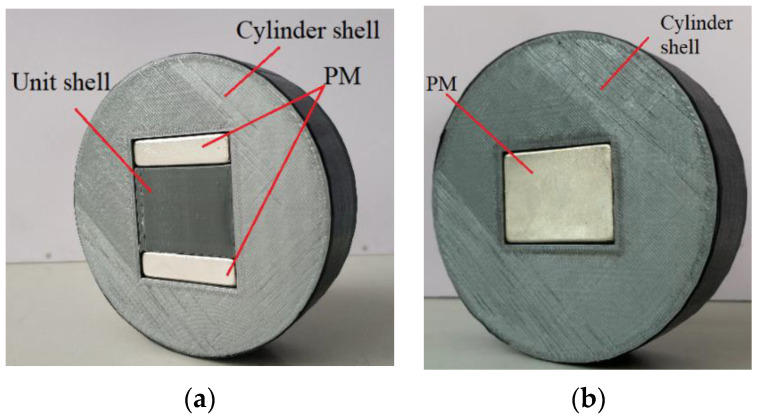
Configuration of two types of test samples. (**a**) Type A; (**b**) Type B.

To study the effect of the magnetic field on the STL of the unit filled with MRF (MRF unit), two types of test samples, shown in Figure 1, were designed and fabricated. Each test sample consisted of a cylindrical shell, unit shell, several permanent magnets (PMs), and the MRF.

The materials of the cylinder shell and unit shell were acrylonitrile butadiene styrene plastic, and the shells were fabricated by three-dimensional (3D) printing technology. The cylinder shell was used to mount the unit shell and the PMs. To be installed smoothly into the impedance tube, the diameter of the cylinder shell should be slightly smaller than the inner diameter of the impedance tube. The unit shell was a 30 × 30 × 40 mm^3^ rectangular unit with a lid, and the MRF was filled in it. The MRF used in this study was MRF−J25T provided by Chongqing Materials Research Institute. The MRF specifications are tabulated in Table 1. The unit shell and PMs were embedded in the cylinder shell according to the arrangements shown in Figure 1. The clearance between the unit shell, PMs, and cylinder shell was sealed using silicone grease. Figure 1a shows the perpendicular configuration for the Type A test sample; the magnetic field direction provided by the PMs is perpendicular to the direction of the sound wave propagation. Figure 1b shows the parallel configuration for the Type B test sample; the magnetic field direction provided by the PMs is parallel to the direction of the sound wave propagation.

Properties of the materials used in the test samples are shown in Table 2.

In this study, the magnetic field was provided by the NdFeB PMs, and the adjustment of the magnetic field intensity was actualized by changing the total thickness of the PMs. The dimensions of the PM were 40 × 30 mm^2^, and they had two basic thicknesses of 5 and 10 mm. Different magnetic field intensities can be obtained by combining PMs of different thicknesses. For convenience, we used the total thickness of the combined PMs to represent the corresponding magnetic field intensity. The total thicknesses of different combinations of PMs and the type code of the test samples under different magnetic field intensities are listed in Table 3.

**Figure 2:**
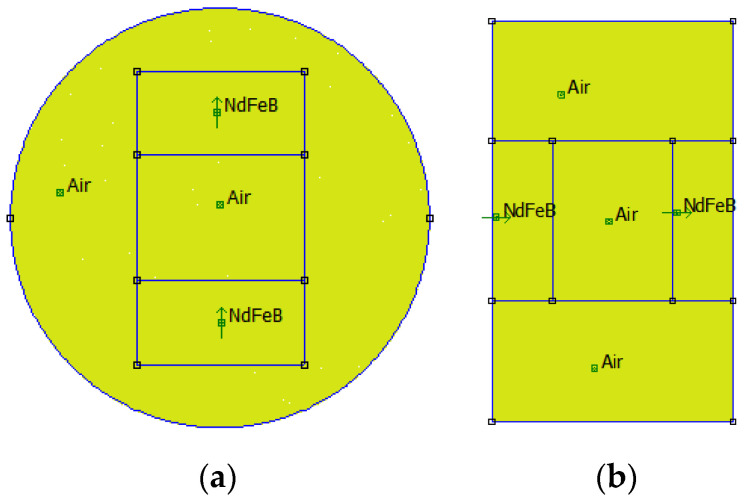
Finite element models for two test samples. (**a**) Type A−20 (Front View); (**b**) Type B−15 (Side View).

**Figure 3:**
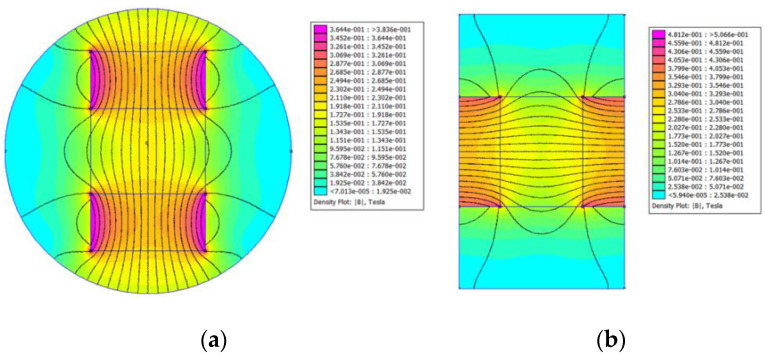
Distribution of the magnetic field for test samples without MRF unit. (**a**) Type A−20; (**b**) Type B−15.

**Figure 4:**
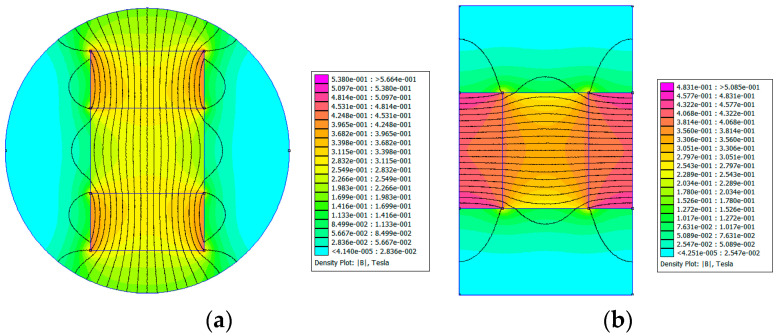
Distribution of the magnetic field for test samples with MRF unit. (**a**) Type A−20; (**b**) Type B−15.

To gain a better understanding of the distribution of the magnetic field intensity, a magnetostatic analysis of the PMs in the test samples was conducted using the finite element method. Open-source finite element software, FEMM 4.2, was utilized in this study. Type A−20 and Type B−15 test samples were considered as two examples, and their finite element models are shown in Figure 2. In the models, the default Neumann boundary condition was used, and a triangular mesh was generated. The minimum angle constraint sent to the mesh generator was 30°. The solver precision parameter was set to 1 × 10^−8^. The mesh sizes of the air area and magnetic area were set to 0.2 mm and 0.1 mm, respectively. The number of nodes for the Type A−20 and Type B−15 test samples were 348,488 and 264,001, respectively. Figure 3 and Figure 4 show the distribution of magnetic field intensity for test samples without and with MRF unit, respectively. It can be seen that the magnetic field in the gap between PM poles is approximately uniform and the distribution of magnetic field for the test samples considering the MRF permeability inserted is more uniform. Therefore, the magnetic field intensity applied to the MRF can be expressed as the average of the magnetic field intensities measured in the gap between PM poles.

To obtain a more accurate value of the magnetic field intensity applied to the MRF, the magnetic field intensities at the middle and two ends of the gap between the PM poles were measured using a WT−4B digital Teslameter. The magnetic field intensity at each position was measured three times, and the average of the three data points was taken as the measured value at this position. The average of the measurements from the three positions was considered as the magnetic field intensity corresponding to this type of PM combination. The magnetic field intensities provided by different PM combinations are listed in Table 4. From Figure 3 and Table 4, we can see that the simulated magnetic field intensities are in excellent agreement with the experimental results.

It is evident that except for the MRF, the other three parts of the test sample, namely, the cylinder shell, PMs, and unit shell, contribute to the STL of the test sample. To eliminate the influence of these parts on the STL, other test samples, including the unit shell filled with nothing (named air unit) and the unit shell filled with water (named water unit), were also prepared and used in the experiments.

## 3. Experimental Study

### 3.1. Description of the Experimental Setup

There are two methods for measuring the STL of materials or structures: the two-room method and impedance tube method. The two-room method involves two adjacent rooms with suitable acoustic characteristics in a large laboratory, and the STL of test sample was characterized by taking measurements in the two rooms [28]. The impedance tube method involves a set of rigid tubes, where sound is internally guided and propagates along the tube axis. Compared to the two-room method, the impedance tube method is cheaper and has fewer space requirements [29]. Therefore, the impedance tube method has been widely applied in STL tests of materials and structures [30].

**Figure 5:**
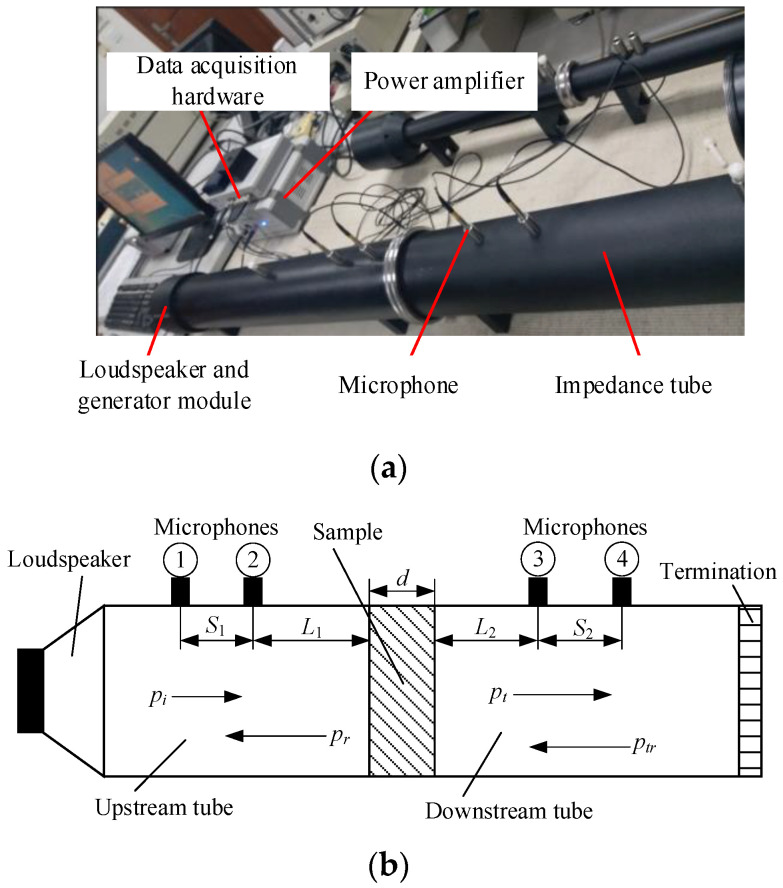
STL test system and test schematic of the impedance tube method. (**a**) STL test system; (**b**) test schematic of the impedance tube method.

In this study, the STL of the test sample was measured using an impedance tube system (BSWA Technology Co., Ltd., Beijing, China) following the ISO 10534-2 standard test procedure. The STL test system shown in Figure 5a consists of impedance tubes (BSWA SW422 Type 510045), four microphones (Type MPA416), a power amplifier, loudspeaker, generator module, data acquisition hardware, and measurement software. The test frequency of the signal generator ranged from 10 to 20 kHz. The power amplifier had an input sensitivity of 350 mV and an output power of 200 W, and the frequency response ranged from 6 Hz to 78 kHz. A test schematic of the impedance tube with four microphones is demonstrated in Figure 5b. The loudspeaker was mounted at one end of the upstream tube, and the sound source was considered as a plane wave source. An acoustic termination occurs at the end of the downstream tube. Test samples were installed between the upstream and downstream tubes. The plane sound wave travels in the upstream tube, arrives at the surface of the test sample, and then a part of the sound wave is reflected, and a part of the wave is transmitted through the test sample into the downstream tube. In Figure 5b, *p_i_* and *p_t_* represent the incident and transmitted sound pressures, respectively; *p_r_* and *p_tr_* represent the sound pressures reflected by the test sample and acoustic termination, respectively; *L*_1_ and *L*_2_ represent the distances from the microphones to the surfaces of the test sample; and *S*_1_ and *S*_2_ represent the distances between two microphones. The thickness of the test sample is denoted by *d*.

The sound transmission coefficient *t_p_* of the test sample can be obtained by

(1) $$t_{p}=\frac{p_{t}}{p_{i}}=\frac{p_{3}e^{jkS_{2}}-p_{4}}{p_{1}-p_{2}e^{-jkS_{1}}}\frac{\sin(kS_{1})}{\sin(kS_{2})}e^{jk(L_{1}+L_{2})}$$

where *k* is the number of waves.

According to the definition of STL, it can be expressed as

(2) $$STL=-20\mathrm{lg}\left|t_{p}\right|$$

### 3.2. Experimental Procedures

For the STL test using the impedance tube method, the installation mode of the test sample and the quality of sealing between the test sample and the impedance tube have a significant impact on the test results; therefore, the size of the test sample should be as close as possible to the size of the impedance tube. After the test sample is installed in the impedance tube, a sealing operation is required to avoid sound leakage and inaccurate measurement results. In this experiment, the measurements were performed in the frequency range of 64–1600 Hz, and an impedance tube with a diameter of 100 mm was adopted. As stated before, the diameter of the test sample designed in this study is slightly smaller than the diameter of the impedance tube; therefore, the sealing operation must be performed. Before the test sample was installed in the impedance tube, silicone rubber was applied evenly along the thickness side of the sample. After the test sample was installed in the impedance tube, the clearance between the impedance tube and test sample was checked, and excess residual silicone rubber was removed. If clearance between the impedance tube and the test sample still existed, an appropriate amount of silicone rubber was applied until no clearance existed.

The calibration of sensors is a necessary preparation step before experiments [31,32]. Before the experiments were conducted, four microphones inserted in the impedance tube were calibrated using a pistonphone and calibration box. To stabilize the working state, the loudspeaker was operated for 10 min before the experiments. Each experimental run was repeated five times to ensure repeatability and consistency. The final results were the average of five measurements.

## 4. Results and Discussion

In this section, STL of the test samples measured under different magnetic field directions and intensities are presented, and the effects of the magnetic field intensity and direction on the STL are discussed. As mentioned before, for the Type A and Type B test samples, the magnetic field directions were perpendicular and parallel to the direction of sound wave propagation, respectively. The thickness of the PMs was used to represent the magnetic field intensity.

### 4.1. Effect of the Magnetic Field Intensity

**Figure 6:**
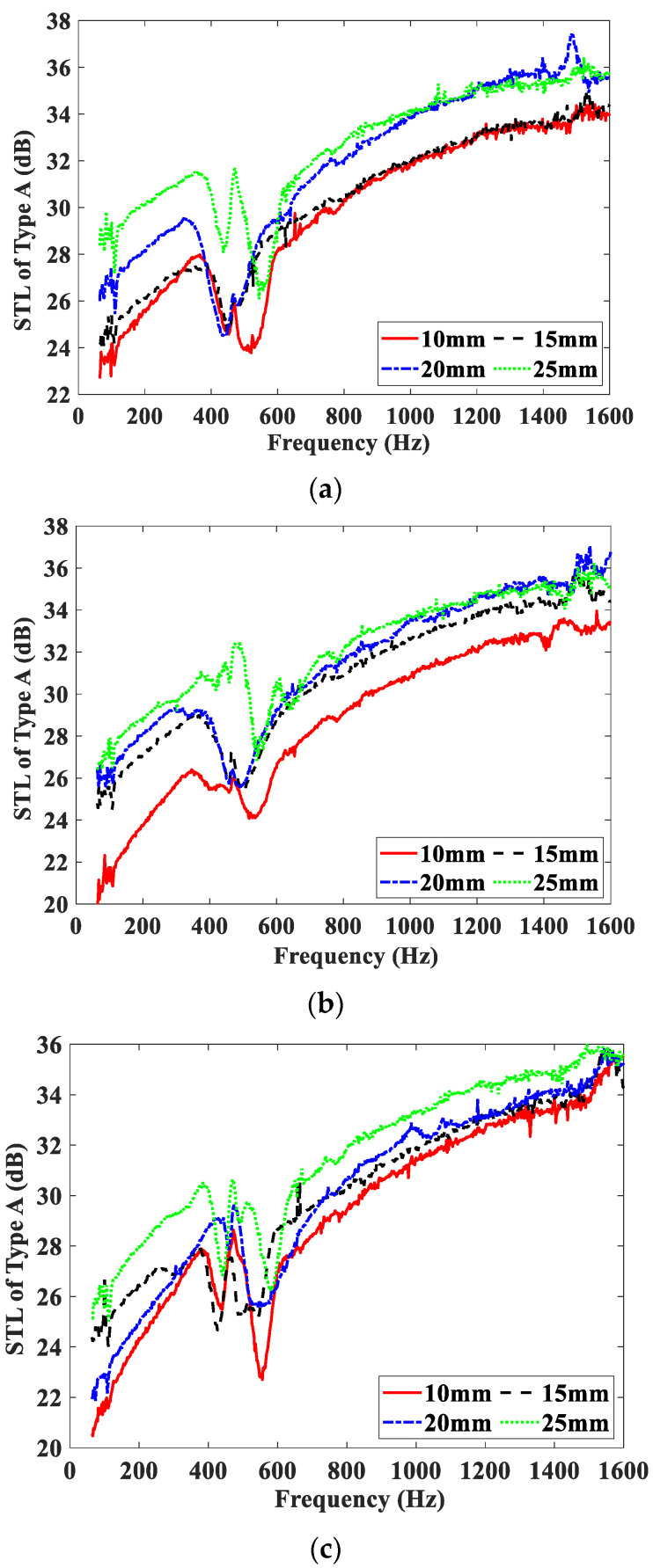
STL curves of Type A test samples under different magnetic field intensities. (**a**) Embedded with MRF unit; (**b**) embedded with air unit; (**c**) embedded with water unit.

Figure 6 shows the effect of the magnetic field intensity on the STL curves of the Type A test samples, and these test samples are embedded with MRF, air, and water units, respectively. The STL curves of the three test samples measured under four different magnetic field intensities are shown in Figure 6a–c. From Figure 6, it can be observed that the STL curves of the Type A test samples have similar varying trends. These curves all show the frequency characteristics of the traditional sandwich panel. The curves cover three regions in the frequency range of concern, namely, the stiffness-controlled, resonance-controlled, and mass-controlled regions. The frequency bands corresponding to the three regions are approximately 64–400 Hz, 400–600 Hz, and 600–1600 Hz, respectively. Comparing Figure 6a–c, it can be seen that the difference among the STL curves of the three test samples embedded with different units is very small, and the STL increases only slightly with the increase of the magnetic field intensity. That means that compared with air and water, the MRF does not show its advantages as an intelligent fluid. The increasing of STL with the magnetic field can be regarded as being mainly caused by an increase in the PM thickness.

**Figure 7:**
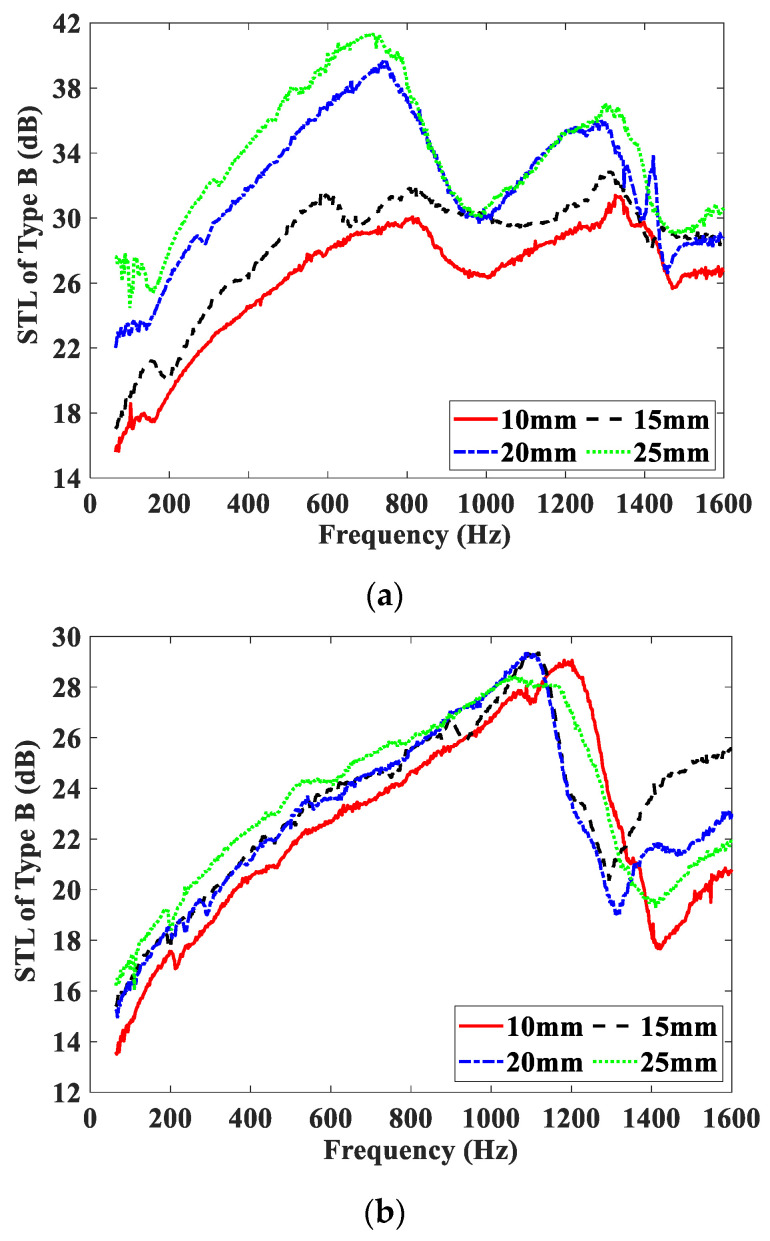

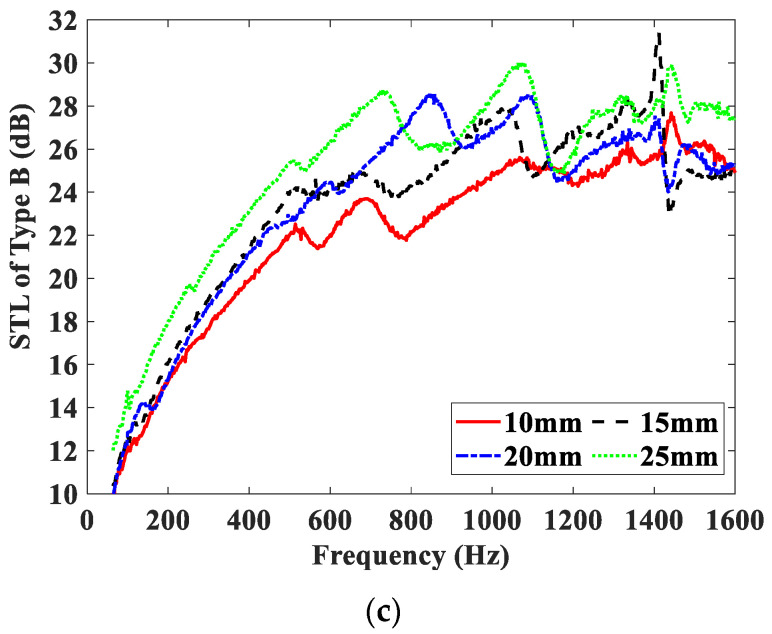
STL curves of Type B test samples under different magnetic field intensities. (**a**) Embedded with MRF unit; (**b**) embedded with air unit; (**c**) embedded with water unit.

Figure 7 shows the effect of the magnetic field intensity on the STL curves of the Type B test samples, and these test samples are embedded with MRF, air, and water units, respectively. The STL curves of the three test samples measured under four different magnetic field intensities are shown in Figure 7a–c. From Figure 7, it can be seen that the varying trends of STL curves of the three test samples are significantly different from those of the Type A test samples. Since the PMs are installed in the propagation direction of the acoustic wave, the STL curves of the type B test samples shift in the upward and higher frequency direction as a whole. For test samples embedded with the air or water unit, the increase of STL with the magnetic field is mainly due to the increase of the PMs’ thickness. However, for test samples embedded with the MRF unit, the STL increases significantly with increasing of the magnetic field intensity at low and middle frequencies, and the increase of STL includes two parts: one is mainly caused by the increase of the PMs’ thickness, the other is mainly caused by the increase of the scattering loss of the sound wave in MRF. When the PMs’ thickness changes from 10 mm to 25 mm, namely, the magnetic field intensity changes from 0.177 T to 0.277 T, and the peak of the STL curves increases by approximately 15 dB. The increase of the magnetic field intensity makes the magnetic chain structure of the MRF thicker and more stable, and the viscosity of the MRF larger, which leads to the increase of energy loss of sound propagation and then results in the increase of the STL. It is obvious that compared with the Type A test sample embedded with the MRF unit, the Type B test sample has better STL-adjusting performance at low and middle frequencies.

### 4.2. Effect of the Magnetic Field Direction

**Figure 8:**
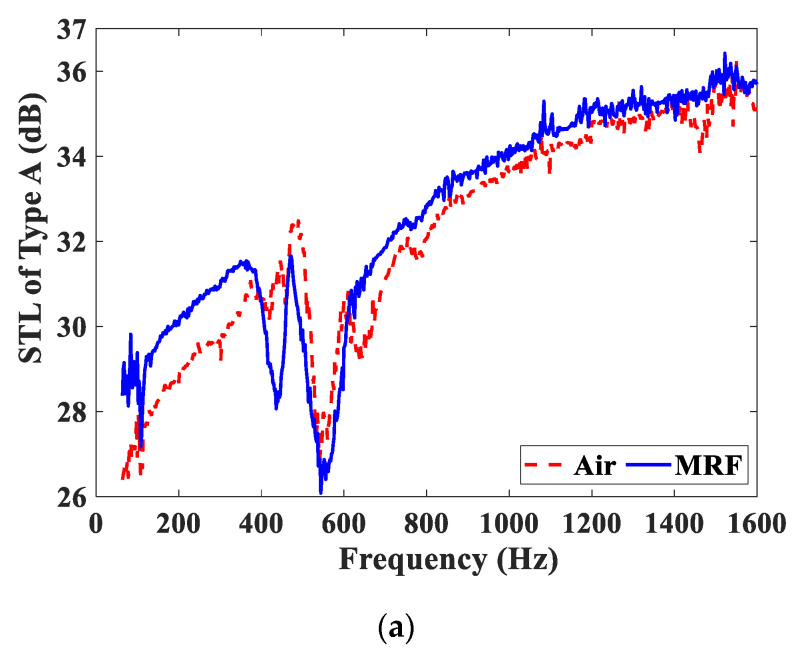

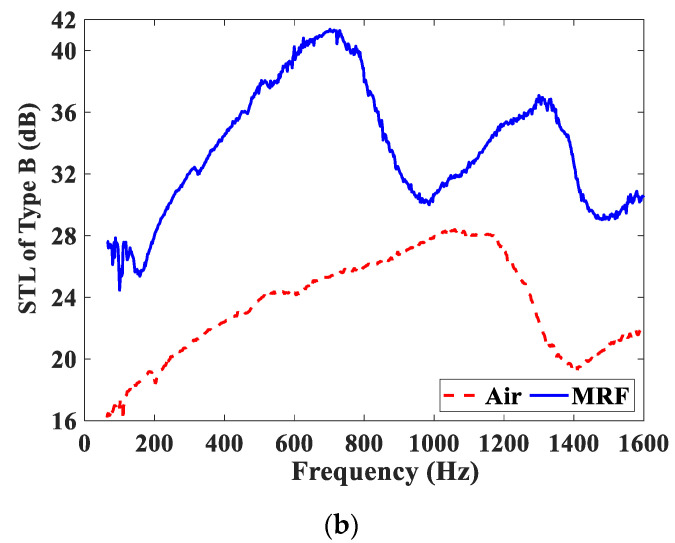
Comparison of STL curves under the same magnetic field intensity. (**a**) Type A−25; (**b**) Type B−25.

For the Type A and Type B test samples, Figure 8 compares the STL curves of the test samples embedded with the MRF and air units under the same magnetic field intensity. As shown in Figure 8a, the STL curve of the Type A−25 test sample embedded with the MRF unit is slightly higher than that of the test sample embedded with the air unit. This means that when the magnetic field direction is perpendicular to the direction of the sound wave propagation, the MRF unit has no apparent advantages in sound insulation performance over other media. In Figure 8b, the STL curve of the Type B−25 test sample embedded with the MRF unit is greatly higher than that of the test sample embedded with the air unit. The STL peak value of the test sample embedded with the MRF unit is greater than 40 dB. Obviously, when the magnetic field is applied parallel to the sound propagation, MRF demonstrates its absolute advantages in acoustic performance compared with the air.

MRFs are heterogeneous media composed of the carbonyl iron powder and carrier fluid. According to the literature [33], the microstructure of MRFs and their evolution in working states are highly complex and influenced by many factors. Before the magnetic field is applied, the suspended iron particles are uniformly distributed in the carrier fluid and the MRF is an isotropic suspension at this time, and the propagation of sound waves in the MRF is similar to that in the ordinary suspension. After the magnetic field is applied, the suspended particles start to be magnetized and become dipoles. The dipoles attract and move each other due to the action of the magnetic dipole moment parallel to the magnetic field direction, resulting in the suspended particles aligning to form a chain structure. Under the action of the magnetic field, the chain structure is further arranged along the magnetic field direction to form an ordered structure, which makes the viscosity and the internal structure of the MRF change significantly.

When the magnetic field direction is perpendicular to the direction of sound wave propagation, the MRF chain structure in the direction of the magnetic field greatly reduces the suspended particles on the propagation path of the sound wave, thereby reducing the total scattering loss of the sound wave. Therefore, the Type A test sample embedded with the MRF unit cannot show obvious advantages in the sound insulation performance over the samples embedded with the air or water unit. When the magnetic field direction is parallel to the direction of sound wave propagation, the MRF chain structure in the direction of the magnetic field greatly increases the suspended particles on the propagation path of the sound wave, which can be regarded as the increase of the volume fraction of the particles, thereby increasing the total scattering loss of the sound wave. Therefore, compared with the Type B test samples embedded with the air or water units, the Type B test sample embedded with the MRF unit has higher STL.

Summarily, the ordered chain structure or column structure makes the MRF anisotropic. When the sound wave passes through the MRF from different directions, its velocity and wave amplitude will vary, resulting in the different scattering loss of the sound wave. Therefore, significant changes in the internal structure of MRF have a significant impact on the propagation characteristics of sound waves. When the magnetic field direction is parallel to the direction of the sound wave propagation, the MRF unit can provide excellent sound insulation performance at low and middle frequencies.

The experimental error is also a matter of concern [34,35]. Undeniably, some errors were introduced in the above test results because the change in the magnetic field intensity was actualized by changing the PMs in this study. To assess the repeatability error, half of the difference between the highest and lowest measured values was calculated, and the results changed but remained in the range of 4–6% for the change in STL. In addition, the above experimental results are in good agreement with previously published works [18,25].

## 5. Conclusions and Future Work

The influence of the magnetic field intensity and direction on the STL of a unit filled with MRF were investigated in this study. The following conclusions were drawn:

(1) The simulated distribution of the magnetic field intensity provided by the PMs showed that the magnetic field applied to the MRF was uniform.
(2) The experimental results for the STL demonstrate that the magnetic field direction has a significant influence on the sound insulation performance of the MRF unit. When the magnetic field direction is perpendicular to the sound propagation direction, the MRF has no apparent advantages in terms of sound insulation performance over other media.
(3) When the magnetic field direction is parallel to the sound propagation direction, the MRF unit can obtain a significant STL at the low and middle frequencies. In addition, the STL of the MRF unit significantly increased with increasing magnetic field intensity. When the magnetic field intensity changed from 0.177 T to 0.277 T, the peak of the STL curves increased by approximately 15 dB (up to 60% variation). This means that MRFs can be used as a core material of sandwich structures to actively control their sound insulation performance at low and middle frequencies.

A possible extension of this work would be to explore an appropriate strategy for controlling the magnetic field to maximize the STL. The acoustic properties of MRFs depend largely on their microstructures under the action of magnetic fields. However, the microstructures of MRFs and their evolution in working states are highly complex. Another extension would be to further investigate the theoretical and simulation models considering the factors influencing the microstructure of the MRFs.

## Figures and Tables

**Table 1:** Specifications of the MRF.

Based Fluid	Density	Particles Packing (vol.)	Viscosity (20 °C) (*γ* = 10/s)	Shear Stress (B = 0.5 T)	Operating Temperature
Hydrocarbon	2.65 g/cm^3^	25%	0.8 Pa.s	>50 kPa	−40 °C to 130 °C

**Table 2:** Properties of the material used in the test samples.

Material	Density	Poisson’s Ratio	Elasticity Modulus
ABS	1020 kg/m^3^	0.39	2 GPa
PM	6000 kg/m^3^	0.24	0.15 GPa

**Table 3:** Total thickness of PMs and the corresponding type code.

Total Thickness (mm)	Type Code	
10	Type A−10	Type B−10
15	Type A−15	Type B−15
20	Type A−20	Type B−20
25	Type A−25	Type B−25

**Table 4:** Magnetic field intensities applied to test samples.

Number	Intensity (T)	Number	Intensity (T)
Type A−10	0.142	Type B−10	0.177
Type A−15	0.173	Type B−15	0.227
Type A−20	0.193	Type B−20	0.264
Type A−25	0.223	Type B−25	0.277

## Data Availability

Not applicable.
